# Seasonal variations in the diagnosis of childhood cancer – reply

**DOI:** 10.1054/bjoc.2000.1377

**Published:** 2000-08-16

**Authors:** J A Ross, R K Severson

**Affiliations:** Division of Epidemiology/Clinical Research, Department of Paediatrics, University of Minnesota Medical School, Minneapolis, USA


					700 Letter to the Editor
British Journal of Cancer (2000) 83(5), 699-700 (c) 2000 Cancer Research Campaign
In the meantime, we suggest that all studies reporting on season-
ality should utilize individual patient data, provide estimates of the
day of the peak date with a CI and report on magnitude.
D Machin1,2
and F Gao1
1
Division of Clinical Trials & Epidemiological Sciences, National
Cancer Centre, 11 Hospital Drive, Singapore 169610; 2
School of
Health and Related Research, University of Sheffield, Northern
General Hospital, Herries Road, Sheffield S5 7AU, UK
REFERENCES
Fisher NI (1993) Statistics of Circular Data. Cambridge University Press:
Cambridge
Machin D and Chong SF (1998) On the detection of the seasonal onset of disease. J
Epid Biostats 3: 385-394
Machin D and Chong SF (1999) Seasonal variations in the presentation and growth
of thyroid cancer. Br J Cancer 79: 1626-1627
Ross JA, Severson RK, Swensen AR, Pollock BH, Gurney JG and Robison LL
(1999) Seasonal variations in the diagnosis of childhood cancer in the United
States. Br J Cancer 81: 549-553
Westerbeek RMC, Blair V, Eden OB, Kelsey AM, Stevens RF, Will AM, Taylor GM
and Birch JM (1998) Seasonal variations in the onset of childhood leukaemia
and lymphoma. Br J Cancer 78: 119-124
We thank Machin and Gao for their interest in our paper exploring
seasonal variations in the diagnosis of childhood cancer. As they
note, different statistical tests have been used to evaluate seasonal
variations, and although these tests are statistically valid, they
might not make full use of the data. We appreciate Machin and
Gao's application of the Fisher method to our data. The Fisher
method does provide a bit more precision than Roger's
test (including an estimate of the strength of the peak). The
conclusions of our paper, however, remain. Moreover, it is unclear
how clinically useful it is to identify a peak that occurs on a
specific day rather than within a season. Finally, we agree with
Machin and Gao that a formal meta-analysis of all papers
describing seasonality in the diagnosis of childhood cancer may be
helpful; we look forward to when this analysis is conducted.
JA Ross and RK Severson
Division of Epidemiology/Clinical Research,
Department of Paediatrics,
University of Minnesota
Medical School
Minneapolis, USA
Seasonal variations in the diagnosis of childhood cancer
- reply
doi: 10.1054/ bjoc.2000.1377, available online at http://www.idealibrary.com on

				

